# LncRNA SCIRT is downregulated in atherosclerosis and suppresses the proliferation of human aortic smooth muscle cells (HAOSMCs) by sponging miR-146a in cytoplasm

**DOI:** 10.1186/s13019-021-01700-x

**Published:** 2021-11-06

**Authors:** Wenhui Gao, Rong Li, Jingjing Yu, Xijie He, Duo Xu, Hai Zhong, Wenwen Dong, Hanbin Cui

**Affiliations:** 1 Department of Cardiovascular, HangzhouBay Hospital, Binhaier Road, HangzhouBay County, Ningbo, 315000 Zhejiang Province People’s Republic of China; 2 Department of Pathology, No.2 Hospital Yinzhou County, Ningbo, Zhejiang Province People’s Republic of China; 3 Department of Cardiovascular, No. 1 Hospital, Ningbo, Zhejiang Province People’s Republic of China; 4 Department of Cardiology, Hospital of Cilin, Ningbo, Zhejiang Province People’s Republic of China; 5 Department of Thoracic Surgery, No.2 Hospital Yinzhou County, Ningbo, Zhejiang Province People’s Republic of China

**Keywords:** SCIRT, miR-146a, Atherosclerosis, Proliferation

## Abstract

**Background:**

SCIRT has been characterized as a key player in cancer biology, while its role in other human diseases is unclear. This study explored its role in atherosclerosis, with a specific focus on its interaction with SCIRT and miR-146a.

**Methods:**

The expression of SCIRT and miR-146a in atherosclerosis-affected tissues and healthy tissues from 56 atherosclerosis patients were analyzed by RT-qPCR. The expression of SCIRT in nuclear and cytoplasm samples was detected by RNA fractionation assay. The direct interaction between SCIRT and miR-146a was detected by RNA pull-down assay. SCIRT and miR-146a were overexpressed in human aortic smooth muscle cells (HAOSMCs) to study the crosstalk between them. The role of SCIRT and miR-146a in the proliferation of HAOSMCs was analyzed with BrdU assay.

**Results:**

SCIRT was downregulated by atherosclerosis, while miR-146a was upregulated by atherosclerosis. SCIRT was detected in both cytoplasm and nuclear samples, and it directly interacted with miR-146a. In HAOSMCs, overexpression of SCIRT and miR-146a did not affect the expression of each other. Interestingly, SCIRT suppressed the proliferation of HAOSMCs and reduced the enhancing effects of miR-146a on cell proliferation.

**Conclusion:**

Therefore, SCIRT is downregulated in atherosclerosis and it suppresses the proliferation of HAOSMCs by sponging miR-146a in cytoplasm.

## Introduction

As a common chronic disease, atherosclerosis is caused by the formation of atheromatous plaque, which is mainly composed of cholesterol, fats and other substances [1, 2]. The plaques block blood flow and may burst to trigger blood clots [1, 2]. Atherosclerosis at early stages shows no symptoms [3]. Once severe conditions have developed, depending on the arteries that are affected, atherosclerosis may cause kidney problems, peripheral artery disease, stroke and coronary artery disease [4, 5]. Atherosclerosis can usually be treated with statins and fibrates to reduce cholesterol, ACE inhibitors to prevent the narrowing of arteries, and beta-blockers to reduce blood pressure [6, 7]. With active treatment and the changing in lifestyle, early atherosclerosis may be reversible, while atherosclerosis in advanced stages still cannot be treated efficiently.

Various risk factors, such as tobacco smoking, unhealthy diet, diabetes and hypertension, have been characterized for atherosclerosis, however, the exact cause of atherosclerosis is unclear [8]. Recent studies have characterized a considerable number of molecular factors involved in atherosclerosis [9, 10]. Some molecular factors with critical roles in the initiation and progression of atherosclerosis are considered as potential targets for the targeted atherosclerosis therapy, which regulate the expression of disease-related genes to improve atherosclerosis [9, 10]. Long non-coding RNAs (lncRNAs) and microRNAs (miRNAs) lack the capacity of protein-coding, but participate in the regulation of protein synthesis to mediate human diseases, including atherosclerosis [11–13]. Therefore, these ncRNAs are potential targets to treat atherosclerosis. SCIRT plays critical roles in breast cancer, where it counteracts EZH2 and SOX2 to suppresses cancer cell self-renewal mechanism, thereby inhibiting cancer progression [14]. However, its role in other human diseases is unknown. We predicted that SCIRT could interact with miR-146a. MiR-146a can regulate the proliferation of vascular smooth muscle cells [15], which participate in atherosclerosis [16]. We therefore investigated the crosstalk between SCIRT and miR-146a in atherosclerosis. We further explored the interaction between SCIRT and miR-146a in atherosclerosis as well.

## Methods

### Study populations

This study enrolled a total of 56 atherosclerosis patients and 56 healthy controls who were admitted to HangzhouBay Hospital from May 2018 to November 2020. The Ethics Committee of this hospital approved this study. The diagnosis of atherosclerosis was performed based on the following criteria: coronary diameter is 1.5 times bigger that of the diameter of adjacent segment or the diameter of the original caliber. Patients with other severe diseases were excluded. Patients with initiated therapy were also excluded. All patients provided the written form informed consent. The clinical features of both groups of participants were shown in Table 1.

**Table 1:** Clinical features of two groups of participants

	Control (n = 56)	Atherosclerosis (n = 56)
Male	32	32
Female	24	24
Age (year)	58.67 ± 12.11	59.12 ± 11.46
BMI	22.51 ± 2.73	23.69 ± 4.23
Hypertension	32	16**
Smoker	32	30
Diabetes	12	11
WBC count, 109/l	6.19 ± 2.09	6.34 ± 2.77
HDL-C (mmol/l)	0.89 ± 0.42	0.38 ± 0.16**
LDL-C (mmol/l)	2.72 ± 1.12	4.29 ± 0.73**
TG (mmol/l)	1.43 ± 0.38	2.01 ± 0.71**
TC (mmol/l)	3.34 ± 1.21	4.78 ± 1.34**
CR (mmol/l)	97.34 ± 13.55	96.13 ± 13.19
UA (mmol/l)	273.34 ± 48.67	272.34 ± 38.43

**p < 0.01

### Plasma preparations

All participants were fasted for at least 12 h. After that, extraction of blood from elbow vein was performed, followed by transferring blood samples to EDTA tubes. Plasma separation was performed through 10 min centrifugation at 1200*g* at 4 °C. EP tubes (DNase-free and RNase-free) were used to store plasma samples prior to the subsequent analyses.

### Human aortic smooth muscle cells (HAOSMCs)

HAOSMCs (Cat # 354-05A, Sigma-Aldrich) were used in all in vitro cell experiments. FBS was added to DMEM (Gibco) to reach a final concentration of 10%, and this mixture was used to cultivate HAOSMCs at 37 °C with 5% CO_2_ and 95% humidity.

### Cell transfections

Overexpression assays of both SCIRT and miR-146a were performed in HAOSMCs. To overexpress SCIRT and miR-146a, HAOSMCs were transfected with either mimic of miR146a and/or SCIRT. Neon transfection device (Thermo Fisher Scientific) was used to perform all transfection assays. The dosage of miRNA and vector was 10 µg and 40 nM miRNA for 10^6^ cells, respectively. To perform NC assays, transfections of empty vector or NC miRNA were included. To perform control (C) assay, cells without transfections were cultivated until the end of transfections.

### Preparation of RNA samples

Total RNAs were isolated with NucleoSpin RNA Plus (Takarabio). DNA digestion was carried out with DNase I (RiboBio). RNA integrity and concentrations were determined using Bioanalyzer. All samples showed an RIN value higher than 8.0.

### RT-qPCRs

With 3000 ng total RNAs as template, cDNA samples synthesized by reverse transcription. qPCRs were then performed to determine the expression levels of SCIRT and miR-146a with 18S rRNA and U6 as the internal control, respectively. The 2^−ΔΔCT^ method was used to normalize the Ct values of SCIRT and miR-146a to that of the corresponding endogenous control.

### Subcellular fractionation assay

Cell fractionation buffer with NP40 was used to perform cell lysis in HAOSMCs. Cell lysis was performed on ice for 10 min. After that, nuclear and cytoplasm fractions were separated by centrifugations at 1200*g* for 15 min. The fraction of nuclear was further subjected to nuclear lysis with nuclear lysis buffer. NE-PER Nuclear and Cytoplasmic Extraction Reagents (Thermo Fisher Scientific) were used to complete all above-mentioned steps. After that, RNA isolations were performed on both fractions, followed by reverse transcriptions and PCRs to amplify SCIRT. PCR products were separated on 1.5% agarose gels. Following ethidium bromide (EB) staining, images were collected using MyECL imager (Bio-Rad).

### RNA pull-down assay

In vitro transcriptions and biotinylation were performed using T7 RNA transcriptase (Invitrogen) to prepare biotinylated miR-146a (Bio-miR-146a) or NC miRNA (Bio-NC). HAOSMCs were transfected with two miRNAs, followed by cell cultivation using fresh medium. After cell lysis, streptavidin beads were used to pull down the complex. RNA isolations were performed on pull-down samples, followed by RT-qPCRs to determine the expression levels of SCIRT.

### BrdU cell proliferation assay

BrdU incorporation reflects DNA synthesis. In this experiment, incorporation of BrdU was determined to reflect cell proliferation. In brief, HAOSMCs were cultivated for 48 h, followed by incubation with BrdU (10 mg/ml) for another 2 h. After that, anti-BrdU-antibody (peroxidase-coupled, Sigma-Aldrich) was used to incubate with the cells at room temperature for 60 min. After PBS washing, further incubation with peroxidase substrate was carried out for 30 min. Finally, cell proliferation was analyzed by measuring OD values at 450 nm.

### Statistical analysis

ANOVA Tukey’s test and unpaired t test were used to compare multiple and two independent groups, respectively. *P* < 0.05 was statistically significant.

## Results

### Expression analysis of SCIRT and miR-146a in atherosclerosis

**Fig. 1:**
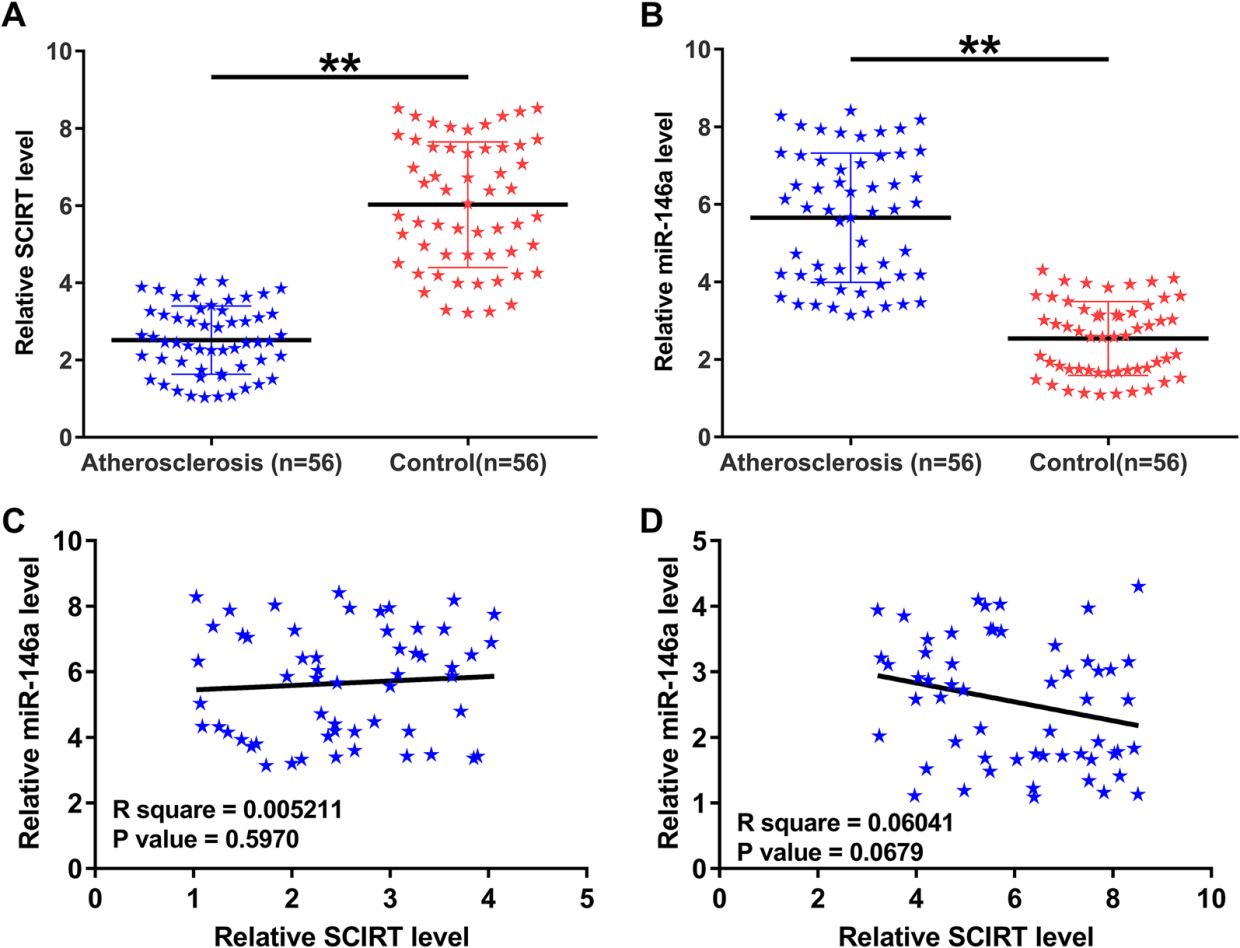
Expression analysis of SCIRT and miR-146a in atherosclerosis. RNA isolations and RT-qPCR were subjected on all plasma samples from both atherosclerosis patients (n = 56) and controls (n = 56) to analyze the differential expression of SCIRT (**A**) and miR-146a (**B**). Pearson correlation coefficient was performed to analyze the correlations between SCIRT and miR-146a across atherosclerosis (**C**) and control (**D**) samples. **p < 0.01

RT-qPCRs were performed to detect the differential expression of SCIRT and miR-146a in plasma samples from both atherosclerosis patients (n = 56) and controls (n = 56). The results showed that SCIRT was significantly downregulated in atherosclerosis (Fig. 1A, *p* < 0.01), while miR-146a was significantly upregulated in atherosclerosis (Fig. 1B, *p* < 0.01). Pearson correlation coefficient analysis revealed that the expression of SCIRT and miR-146a were not correlated across atherosclerosis (Fig. 1C) and control (Fig. 1D) samples.

### The subcellular location of SCIRT in HAOSMCs and the interaction between SCIRT and miR-146a

**Fig. 2:**
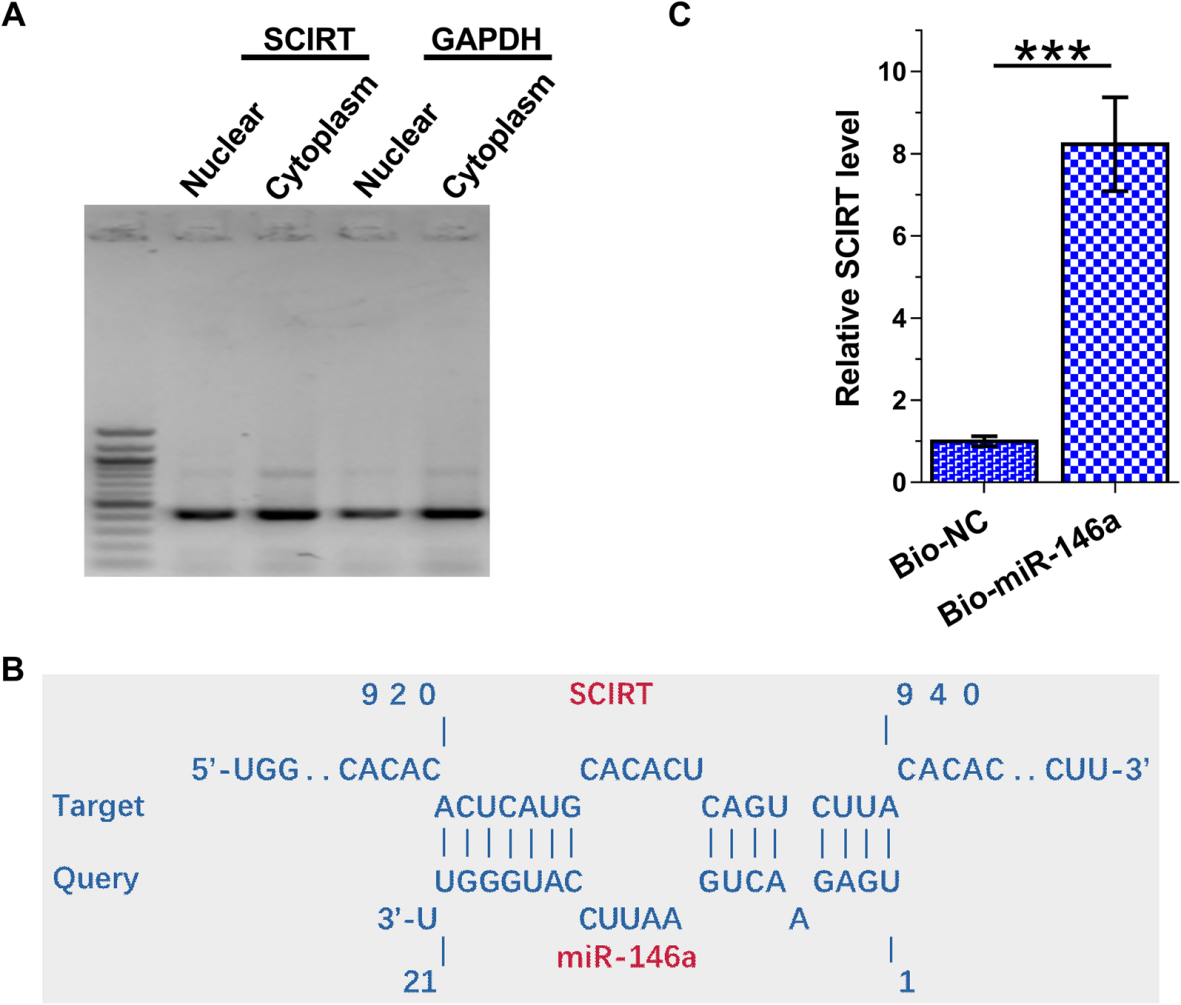
The subcellular location of SCIRT in HAOSMCs and the direct interaction between SCIRT and miR-146a. Subcellular fractionation assay was done to explore the subcellular location of SCIRT in HAOSMCs. Similar to GAPDH, SCIRT was detected in both nuclear and cytoplasm samples of HAOSMCs (**A**). IntaRNA 2.0 was applied to predict the interaction between SCIRT and miR-146a (**B**). The direct interaction between SCIRT and miR-146a was analyzed by RNA pull-down assay (**C**). ***p < 0.001

Subcellular fractionation assay was performed to detect the subcellular location of SCIRT in HAOSMCs. Although GAPDH is a cytoplasm marker and enriched in cytoplasm, it can also be detected in nucleus. In contrast, nearly similar amount of SCIRT was detected in nuclear and cytoplasm samples of HAOSMCs (Fig. 2A). IntaRNA 2.0 was applied to predict the interaction between SCIRT and miR-146a, and the prediction revealed multiple potential base pairings between them (Fig. 2B). RNA pull-down assay was performed to verify the direct interaction between SCIRT and miR-146a. The results showed that expression levels of SCIRT were significantly higher in Bio-miR-146a pull-down sample compared to that in Bio-NC group, suggesting the direct interaction between them (Fig. 2C, *p* < 0.001).

### The roles of SCIRT and miR-146a in regulating the expression of each other

**Fig. 3:**
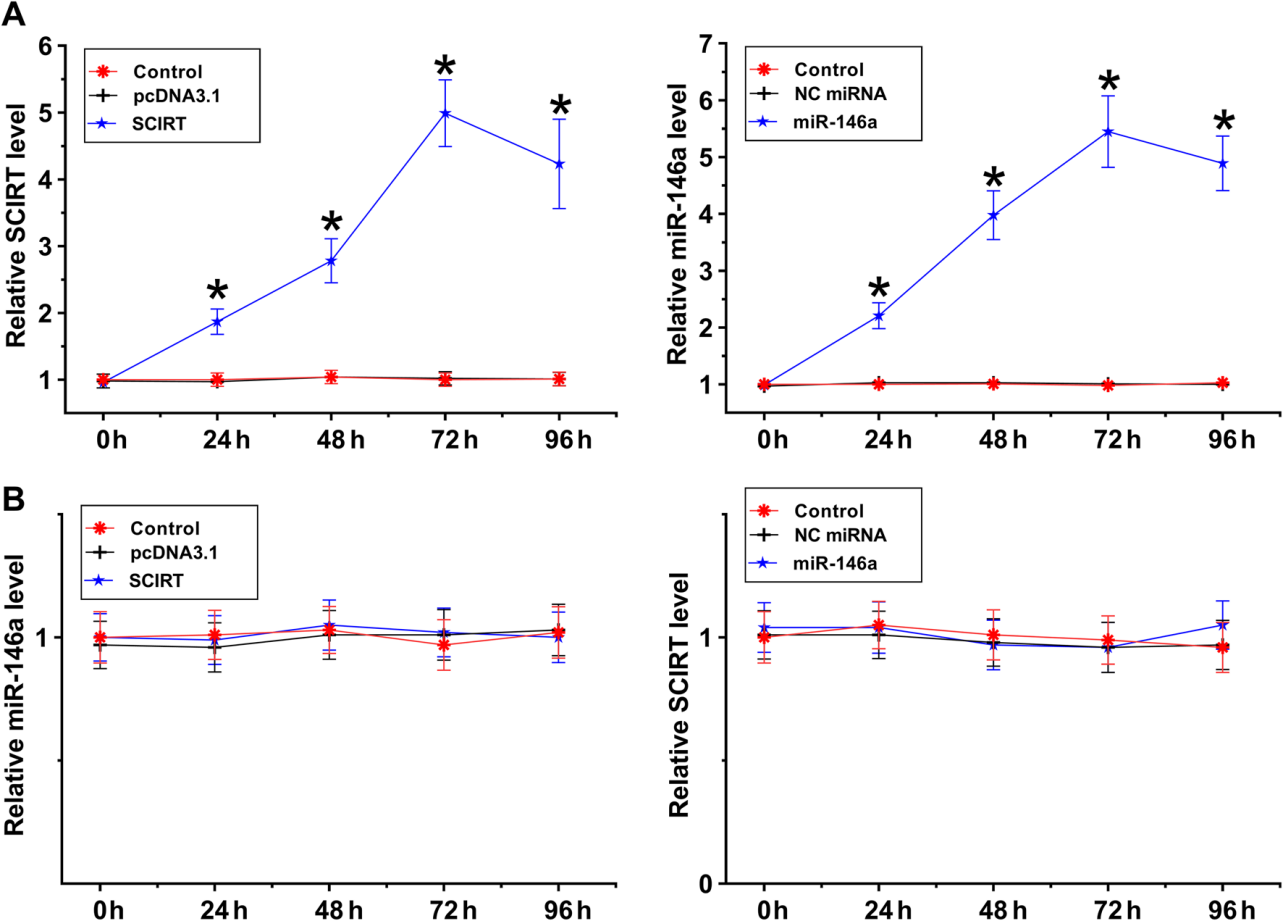
The roles of SCIRT and miR-146a in regulating the expression of each other. To further analyze the crosstalk between SCIRT and miR-146a in atherosclerosis, SCIRT or miR-146a was overexpressed in HAOSMCs. The overexpression of SCIRT or miR-146a was confirmed in HAOSMCs every 24 h until 96 h (**A**). The role of SCIRT and miR-146a in regulating the expression of each other was analyzed by RT-qPCR (**B**). *p < 0.05

To further investigate the crosstalk between SCIRT and miR-146a in atherosclerosis, SCIRT or miR-146a was overexpressed in HAOSMCs. The overexpression of SCIRT or miR-146a was confirmed in HAOSMCs every 24 h until 96 h (Fig. 3A, *p* < 0.05). Surprisingly, overexpression of SCIRT and miR-146a did not regulate the expression of each other (Fig. 3B).

### The role of SCIRT and miR-146a in the proliferation of HAOSMCs

**Fig. 4:**
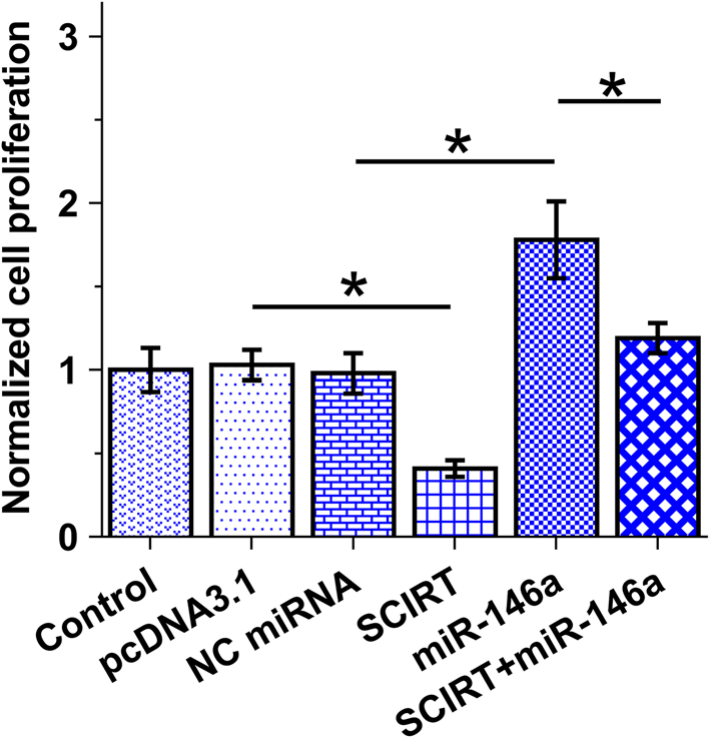
The role of SCIRT and miR-146a in the proliferation of HAOSMCs. BrdU assay was performed to study the role of SCIRT and miR-146a in the proliferation of HAOSMCs. Each experiment was performed in three biological replicate wells. Data were expressed as mean ± SD. *p < 0.05

BrdU assay was performed to study the role of SCIRT and miR-146a in the proliferation of HAOSMCs. The results illustrated that SCIRT could suppress the proliferation of VSMCs, and miR-146a could promote cell proliferation. Moreover, SCIRT inhibited the role of miR-146a in cell proliferation (Fig. 4, *p* < 0.05).

## Discussion

Atherosclerosis is a complex chronic disease with multiple potential factors involved. This study demonstrated the participation of lncRNA SCIRT and miR-146A in atherosclerosis. We showed that SCIRT is downregulated in atherosclerosis and it may sponge miR-146a to suppress the proliferation of HAOSMCs.

To date, the function of SCIRT has mainly been investigated in breast cancer [14]. Although SCIRT is upregulated during the tumorigenesis of breast cancer, it suppresses the cancer cell self-renewal mechanism by counteracting EZH2 and SOX2 to suppress cancer progression [14]. This study is the first to analyze the role of SCIRT in atherosclerosis. We found that SCIRT is downregulated in atherosclerosis. It is known that other clinical disorders and therapies may affect gene expression. Therefore, this study excluded patients complicated with other diseases and the ones with initiated therapy. Vascular smooth muscle cells play prominent roles in atherosclerosis [16]. The aberrant VSMCs proliferation at early stages of atherosclerosis promotes the formation of plaques, which in turn promotes disease progression [16]. In contrast, the increased proliferation of VSMCs prevents fibrous cap rupture, resulting in beneficial effects [16]. In this study we showed that SCIRT could suppress the proliferation of HAOSMCs. Therefore, accurate regulation of the expression of SCIRT at different stages of atherosclerosis may improve disease conditions.

MiR-146a has been reported to form a feedback loop with Krüppel-like factor 4 to regulate the proliferation of HAOSMCs [15]. However, the role of miR-146a in atherosclerosis is unknown. In this study we presented the reduced expression levels of miR-146a in atherosclerosis. Additionally, we confirmed the inhibitory effects of miR-146a on the proliferation of HAOSMCs. To the best of our knowledge, the upstream regulator of miR-146a in atherosclerosis is unknown. The present study showed that miR-146a directly interacted with SCIRT in HAOSMCs, while did not affect the expression of each other. Therefore, SCIRT is unlikely a target of miR-146a. With GAPDH as a cytoplasm marker, our data showed that SCIRT was localized to both nuclear and cytoplasm. It is known that mature miRNAs are only detectable in cytoplasm. We then speculated that SCIRT may sponge miR-146a to suppress its role.

Our study suggested that SCIRT could be a potential target to treat atherosclerosis by regulating cell proliferation. However, this study also has limitations, for example the small sample size and the lack of in vivo assays. The conclusions of this study should be further verified by future studies with bigger sample sizes and animal model experiments.

In conclusion, SCIRT is downregulated in atherosclerosis and its overexpression may suppress the proliferation of HAOSMCs possibly by sponging miR-146a in cytoplasm.

## Data Availability

The datasets used and/or analysed during the current study are available from the corresponding author on reasonable request.

## Abbreviation

HAOSMCs: Human Aortic Smooth Muscle Cells
